# 1273. Activity of Ceftolozane/Tazobactam and Comparators Against Enterobacterales and *P. aeruginosa* Isolates from Pediatric Patients—SMART United States 2017-2019

**DOI:** 10.1093/ofid/ofab466.1465

**Published:** 2021-12-04

**Authors:** Sibylle Lob, Meredith Hackel, C Andrew DeRyke, Kelly Harris, Katherine Young, Mary Motyl, Daniel F Sahm

**Affiliations:** 1 IHMA, Inc., Schaumburg, IL; 2 Merck & Co., Inc., Kenilworth, New Jersey; 3 Merck & Co. Inc, Kenilworth, New Jersey; 4 Merck & Co, Inc, Kenilworth, NJ

## Abstract

**Background:**

Ceftolozane/tazobactam (C/T) is an antipseudomonal cephalosporin combined with a β-lactamase inhibitor approved by FDA and EMA for complicated urinary tract (cUTI) and complicated intraabdominal infections (cIAI), as well as hospital-acquired/ventilator-associated bacterial pneumonia (HABP/VABP) in patients ≥18 years. Clinical trials studying the use of C/T for pediatric patients with cUTI and cIAI are completed, and HABP/VABP pediatric studies are underway. We evaluated the antimicrobial activity of C/T against gram-negative isolates collected from patients ≤17 years in the United States (US) as part of the global SMART surveillance program.

**Methods:**

In 2017-2019, 27 US clinical labs each collected up to 250 consecutive gram-negative pathogens per year. A total of 1336 isolates were collected from pediatric patients. MICs were determined using CLSI broth microdilution and breakpoints. C/T-nonsusceptible Enterobacterales (Ebact) and *P. aeruginosa* were screened for genes encoding β-lactamases.

**Results:**

Among the 944 collected Ebact and 220 *P. aeruginosa* isolates, 40.7% and 76.4%, respectively, were collected from patients with respiratory tract infections, 37.0% and 10.0% from UTI, 13.7% and 8.2% from IAI, and 8.3% and 5.0% from bloodstream infections. The table shows antimicrobial susceptibility of Ebact, *P. aeruginosa*, and select phenotypes. C/T was active against 98% of Ebact, including 50 of 51 and 12 of 13 ESBL-non-CRE phenotype *E. coli* and *K. pneumoniae*, respectively. Among *P. aeruginosa*, C/T was active against 95% of isolates, 9-21 percentage points higher than the comparator β-lactams, and it maintained activity against 71-75% of *P. aeruginosa* isolates nonsusceptible to commonly used β-lactams. Among the 21 C/T-nonsusceptible Ebact, 2 isolates carried KPC and ESBL and 3 isolates carried only ESBL; in 16 isolates no β-lactamases were detected, of which 15 were species with intrinsic AmpC. Among 12 C/T-nonsusceptible *P. aeruginosa*, no acquired β-lactamases were detected, likely indicating chromosomally-mediated resistance mechanisms.

Results Table

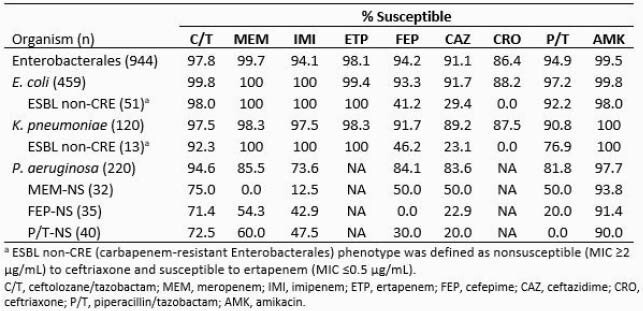

**Conclusion:**

C/T could be a potential new treatment option for US pediatric patients with infections caused by Ebact and *P. aeruginosa*, including resistant phenotypes.

**Disclosures:**

**Sibylle Lob, PhD**, **IHMA** (Employee)**Pfizer, Inc.** (Independent Contractor) **Meredith Hackel, PhD MPH**, **IHMA** (Employee)**Pfizer, Inc.** (Independent Contractor) **C. Andrew DeRyke, PharmD**, **Merck & Co., Inc.** (Employee, Shareholder) **Kelly Harris, PharmD, BCPS**, **Merck & Co. Inc** (Employee) **Katherine Young, MS**, **Merck** (Employee) **Mary Motyl, PhD**, **Merck & Co., Inc.** (Employee, Shareholder) **Daniel F. Sahm, PhD**, **IHMA** (Employee)**Pfizer, Inc.** (Independent Contractor)

